# Supplementary material for: Muscle Function, Body Composition, Insulin Sensitivity and Physical Activity in Adolescents Born Preterm: Impact of Gestation and Vitamin D Status
Source: Nutrients. 2022 Nov 27;14(23):5045. doi: 10.3390/nu14235045 (PMC9736929; doi:10.3390/nu14235045)
Supplement: Supplementary file 1 [file nutrients-14-05045-s001.zip › nutrients-2016015-supplementary.pdf]

Supplementary Information on Magnetic resonance spectroscopy techniques

Magnetic resonance spectroscopy is a technique that can assess mitochondrial oxidative function in muscle and can be viewed as a measure of muscle 'fitness'. The concentration of phosphorus nuclei (^{31}P) in chemically-distinct compounds within muscle, including high energy metabolites such as PCr and ATP, can be quantified using a clinical MRI scanner with modified hardware. The use of a graded exercise apparatus to perform plantar flexion exercises can be used to deplete PCr stores whilst ATP homeostasis is preserved. Cessation of exercise permits oxidative phosphorylation of ATP and recovery of PCr concentrations. The rate of recovery to baseline PCr concentrations within muscle (denoted as $\tau_{½}$PCr) is used as a measure of mitochondrial oxidative function.

Participants were positioned feet-first into the scanner with their left foot placed in a custom-made exercise rig as described previously(1, 2). This allowed plantar flexion movement (0° to 30°) with the foot starting in the anatomically neutral position, isolating the calf muscles during plantar flexion without recruitment of other muscle groups (particularly quadriceps). The rig was used with a force gauge prior to exercise testing to measure the calf muscle maximum voluntary contraction (MVC) for each participant. After MVC recording the participants were given time to recover. An audible cue was provided during the exercise to ensure regular rhythmic flexion, reinforced by a shoulder-tap if they were unable to match the frequency with rhythmic plantar flexion by audible cue alone.

Phosphorus spectra were collected using adiabatic one-Dimensional Image-Selected In-vivo Spectroscopy (1D-ISIS) as previously described. This protocol has been shown to robustly measure mitochondrial oxidative function during recovery from exercise(1, 2).

The data were processed to calculate the various components of oxidative metabolism(3). pH change was calculated by measurement of the changing horizontal displacement of the Pi peak from the PCr peak on the ^{31}P MR spectrum(3).

References

1. Sinha A, Hollingsworth KG, Ball S, & Cheetham T. Improving the Vitamin D Status of Vitamin D Deficient Adults Is Associated With Improved Mitochondrial Oxidative Function in Skeletal Muscle. The Journal of Clinical Endocrinology & Metabolism

2013 **98** E509–E513.

2. Jones DEJ, Hollingsworth KG, Taylor R, Blamire AM, & Newton JL. Abnormalities in pH handling by peripheral muscle and potential regulation by the autonomic nervous system in chronic fatigue syndrome. Journal of Internal Medicine 2010 **267** 394–401.
3. Iotti S, Lodi R, Frassineti C, Zaniol P, & Barbiroli B. In vivo assessment of mitochondrial functionality in human gastrocnemius muscle by 31P MRS. The role of pH in the evaluation of phosphocreatine and inorganic phosphate recoveries from exercise. NMR in biomedicine 1993 **6** 248–253.
